# Endoscopic retrieval of a migrated plastic stent after endoscopic ultrasound-guided hepaticojejunostomy

**DOI:** 10.1055/a-2888-0530

**Published:** 2026-06-30

**Authors:** Tomohisa Iwai, Megumi Tsukamoto, Yusuke Ozaki, Masaki Nishimura, Tomoko Ando, Shigeru Iwase, Shin Maeda

**Affiliations:** 1 Department of Gastroenterology 36993 Fujisawa City HospitalFujisawaKanagawaJapan; 2 Gastroenterology DivisionYokohama City University, School of MedicineYokohamaKanagawaJapan

Endoscopic ultrasound (EUS)-guided biliary drainage has become an established alternative for biliary decompression in patients with surgically altered anatomy or failed endoscopic retrograde cholangiopancreatography (ERCP). 1​ ​2​ However, adverse events such as stent migration remain challenging. 3​ 4​ ​5​ Retrieval of proximally migrated biliary stents can be technically demanding, particularly within the angulated intrahepatic ducts.

**Fig. 1:**
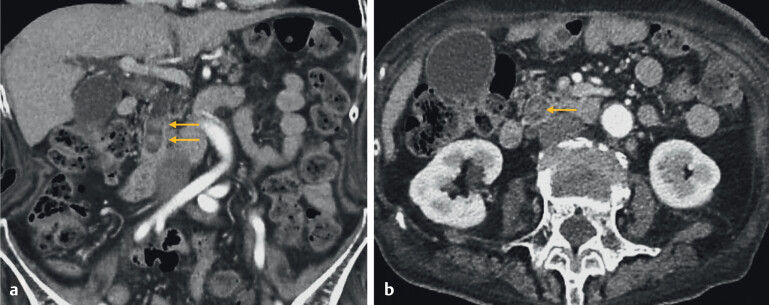
Computed tomography showing multiple common bile duct stones (arrows) in a patient with Roux-en-Y reconstruction after total gastrectomy. ( **a** ) Coronal section. ( **b** ) Axial section.

**Fig. 2:**
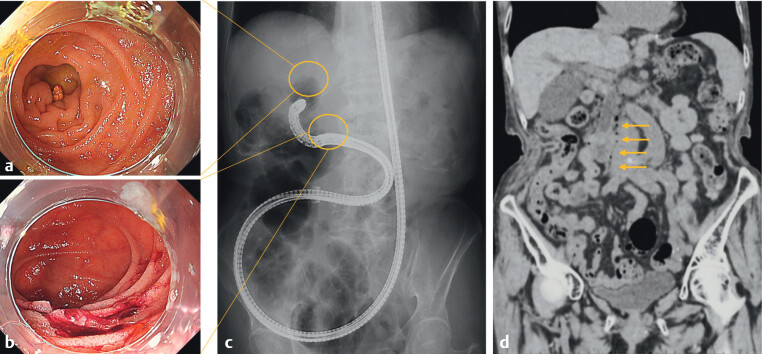
Balloon-assisted ERCP for common bile duct stone removal. ( **a** ) An endoscopic view of the blind end of the duodenum. ( **b** ) Laceration at the inferior duodenal angle. ( **c** ) A fluoroscopic image upon reaching the blind end. ( **d** ) Computed tomography showing retroperitoneal emphysema (arrows) suggesting microperforation.

**Fig. 3:**
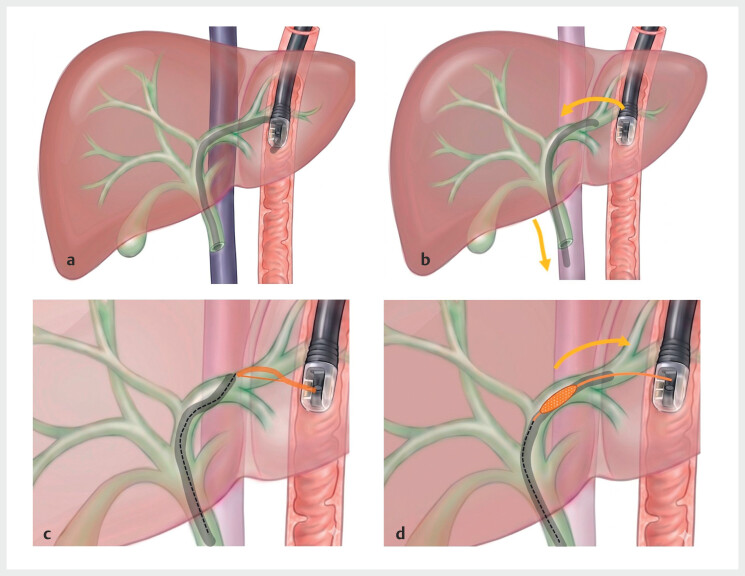
Schema of stent migration into the intrahepatic bile duct and its retrieval after EUS-guided hepaticojejunostomy. ( **a** ) Stent placement into the segment B2 via the jejunal limb. ( **b** ) Complete migration of the stent into the intrahepatic bile duct. ( **c** ) Use of a rotatable sphincterotome to achieve axial alignment with the migrated stent. ( **d** ) Successful retrieval of the stent into the jejunum using a small-caliber balloon.

A 76-year-old woman with Roux-en-Y reconstruction after total gastrectomy was referred for multiple common bile duct stones ( Fig. 1 ). Initial single-balloon enteroscopy-assisted ERCP failed to identify the papilla ( Fig. 2 ). Two months later, the patient developed severe cholangitis. Given the difficult endoscopic access, EUS-guided hepaticojejunostomy (EUS-HJS) was performed. A 7-Fr plastic stent (TYPE-IT; GADERIUS, Tokyo, Japan) was placed from the jejunal limb into the B2 bile duct. Following antegrade stone extraction via the HJS route, re-insertion of the stent was attempted to maintain access. However, the stent migrated completely into the intrahepatic bile duct during deployment, becoming endoscopically invisible from the jejunum. Initial retrieval attempts using grasping forceps, a basket catheter, and a snare were unsuccessful due to the restricted maneuverability within the narrow and angulated intrahepatic bile duct. To overcome this, a rotatable sphincterotome (ENGETSU; KANEKA, Tokyo, Japan) was employed to achieve axial alignment with the migrated stent. This facilitated successful guidewire cannulation into the stent lumen. Subsequently, a small-caliber biliary dilatation balloon (REN; KANEKA, Osaka, Japan) was advanced over the guidewire and inflated inside the stent. Traction with the inflated balloon enabled successful repositioning and subsequent endoscopic retrieval of the migrated stent ( Fig. 3 ).

In conclusion, this “axis-alignment and balloon-traction technique” represents a valuable troubleshooting option for inwardly migrated stents, which is a potential complication in patients with complex anatomy ( Video 1 ).

**Video 1** The successful endoscopic management and retrieval technique for a migrated plastic stent after endoscopic ultrasound-guided hepaticojejunostomy.


Endoscopy_UCTN_Code_TTT_1AR_2AZ
